# Regulation of glucose metabolism by incretins: implications for treatment of type 2 diabetes

**DOI:** 10.1007/s13340-024-00781-y

**Published:** 2024-12-06

**Authors:** Rie Saito, Norio Harada

**Affiliations:** Department of Endocrinology and Metabolism, School of Medical Sciences, University of Fukui, 23-3 Matsuoka Shimoaizuki, Eiheiji-cho Yoshida-gun, Fukui, 910-1193 Japan

**Keywords:** Incretin, GIP, GLP-1, Incretin effect, Insulin secretion

## Abstract

Gastrointestinal hormones that potentiate insulin secretion from pancreatic β-cells are called incretins. Glucose-dependent insulinotropic polypeptide/gastric inhibitory polypeptide (GIP) and glucagon-like peptide-1 (GLP-1) are the major incretins. Incretin-mediated stimulation of insulin secretion (incretin effect) plays an important role in lowering postprandial blood-glucose levels. Incretin effect is reduced under diabetic condition. Several mechanisms have been reported for the reduction of incretin effect. Incretin-related drugs are used worldwide for the treatment of type 2 diabetes. In addition, several new incretin-related drugs have been developed and are expected to be available in clinical practice.

In individuals with normal glucose tolerance, postprandial insulin secretion varies widely in response to the nutrients ingested, and postprandial blood-glucose levels are tightly regulated [1]. Reduced insulin secretory capacity and insulin resistance are involved in the pathogenesis of diabetes [2]. blood-glucose levels are not increased if insulin secretion capacity is sufficient, even in the presence of high insulin resistance. However, fasting and postprandial blood-glucose levels are increased, leading to impaired glucose tolerance and chronic hyperglycemia (the diabetic condition) when insulin secretion capacity is relatively reduced. Westerners and East Asians including the Japanese have increased insulin resistance and decreased insulin secretion as they progress from normal glucose tolerance to impaired glucose tolerance and diabetes. Insulin secretory capacity is markedly decreased in East Asians with diabetes compared to Westerners [3]. In Japanese with impaired glucose tolerance, postprandial blood-glucose levels are more easily elevated than fasting blood-glucose levels [4, 5].

In a study comparing blood glucose and insulin levels during an oral glucose tolerance test (OGTT) and an intravenous glucose tolerance test (IVGTT), insulin secretion was much greater during the OGTT than during the IVGTT, even at the same blood-glucose levels (6). This phenomenon involves gastrointestinal hormones that potentiate insulin secretion from pancreatic β-cells. These gastrointestinal hormones are called incretins: glucose-dependent insulinotropic polypeptide/gastric inhibitory polypeptide (GIP) and glucagon-like peptide-1 (GLP-1) are the major incretins. Incretin-mediated stimulation of insulin secretion (incretin effect) accounts for more than 50% of postprandial insulin secretion and plays an important role in lowering postprandial blood-glucose levels [6].

GIP and GLP-1 have about 40% homology and belong to the glucagon family of peptides, as do secretin and vasoactive intestinal polypeptide (VIP). GIP is secreted from enteroendocrine K cells, which are mainly located in the small intestine, while GLP-1 is secreted from enteroendocrine L cells, which are located in the small intestine and colon [7]. GIP and GLP-1 are secreted in response to food intake and respectively bind to GIP and GLP-1 receptors on pancreatic β-cells (Fig. 1). GLP-1 receptor is also expressed on vagal nerve endings in the portal vein, acting indirectly on insulin secretion via the neural network from the afferent nerves of the hepatic vagus to the centrifugal vagus of the pancreas [8].

**Fig. 1:**
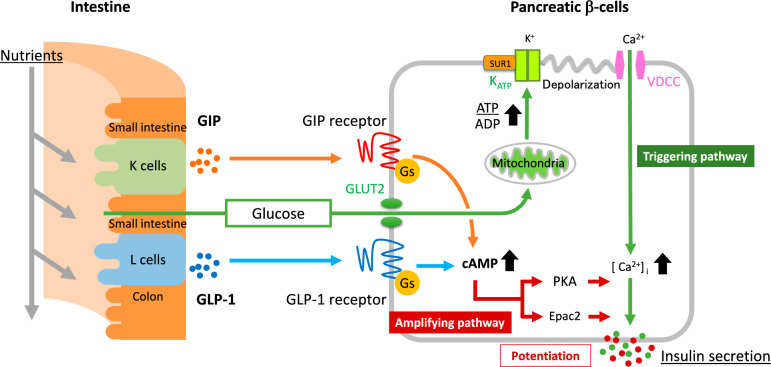
Insulin secretion from pancreatic β-cells. Insulin secretion occurs by metabolizing glucose in β-cells (triggering pathway). GIP and GLP-1 potentiate glucose-dependent insulin secretion via Gs protein signaling (amplifying pathway). *ADP* adenosine-diphosphate, *ATP* adenosine triphosphate,* [Ca*^*2+*^*]*_*I*_ calcium ion influx, *cAMP* cyclic adenosine monophosphate, *Epac2* exchange protein activated by cAMP, *GIP* glucose-dependent insulinotropic polypeptide/gastric inhibitory polypeptide, *GLP-1* glucagon-like peptide-1, *GLUT2* glucose transporter type 2, *Gs* Gs protein, *K*_*ATP*_ KATP channel, *PKA* protein kinase A, *SUR1* sulfonylurea receptor 1, *VDCC* voltage-dependent calcium channel

The GIP and GLP-1 receptors belong to 7-transmembrane G protein-coupled receptors (GPCRs) and mainly couple with Gs protein. GIP and GLP-1 activate adenylyl cyclase, which increases intracellular cyclic adenosine monophosphate (cAMP) (Fig. 1). This increase in intracellular cAMP results in the activation of protein kinase A (PKA) and exchange protein activated by cAMP (Epac2). Activation of both pathways leads to movement of calcium ions from outside the cell via the voltage-dependent calcium channel (VDCC), and from the endoplasmic reticulum into the cytoplasm. As a result, incretin signaling potentiates insulin secretion that is stimulated by glucose metabolism (an amplifying pathway) [9]. The enhancement of insulin secretion by GIP and GLP-1 (incretin effect) is glucose dependent and does not occur at blood-glucose levels below 4.5 mol/L.

In a study of Europeans with type 2 diabetes, in which insulin resistance is the main factor in the development of diabetes, the incretin effect is reduced under diabetic conditions compared to that under normal glucose conditions [6]. Japanese with type 2 diabetes, in whom insulin secretion capacity is mainly impaired during the development of diabetes, also have a decreased incretin effect compared to individuals without diabetes. However, the decrease is smaller in Japanese than that in Europeans [10]. Data from Korean, also East Asians, show no reduction in the incretin effect in individuals with type 2 diabetes, compared to those without [11]. These results indicate that in East Asians, the incretin effect is important for lowering postprandial blood-glucose levels under diabetic conditions as well as under normal glucose conditions.

Incretin secretion occurs after nutrient intake. Plasma incretin levels are known to be influenced by the amount of food consumed and its composition (fats and proteins, etc.). When plasma incretin levels were evaluated after OGTT and a meal-tolerance test in European and Japanese subjects [12, 13], GLP-1 secretion (area under the curve (AUC) of plasma GLP-1 levels) was not significantly different between the two groups. On the other hand, GIP secretion (AUC of plasma GIP levels) was significantly greater in the meal-tolerance test group than in the glucose tolerance test group. In addition, GIP secretion and GLP-1 secretion during OGTT were correlated with various factors such as age, body mass index (BMI), insulin secretion, and AUC of blood-glucose levels, indicating that GIP secretion and GLP-1 secretion were correlated with different factors. Thus, even though both GIP and GLP-1 are incretins, their stimulators and mechanisms of secretion can be different. Previously, it was reported that postprandial GIP secretion is not reduced in Europeans with type 2 diabetes compared to those without, but GLP-1 secretion is reduced in those with type 2 diabetes [14]. These results suggested that the reduced incretin effect in type 2 diabetes is partly due to reduced GLP-1 secretion. A subsequent meta-analysis comparing postprandial incretin secretion in individuals with and without type 2 diabetes concluded that there is no difference in incretin secretion between the two groups [15, 16]. It has been reported that expression of GIP and GLP-1 receptors on pancreatic β-cells is reduced under diabetic conditions [17]. In particular, GIP receptor expression is dramatically reduced compared to GLP-1 receptor expression (Fig. 2A). Furthermore, it has been reported that the main amplifying signaling is altered in β-cells under high glucose conditions [18]. Under these conditions, the amplifying signaling changes from Gs protein-dominance to Gq protein dominance; the GIP receptor activates just the Gs protein, whereas the GLP-1 receptor activates not only Gs protein but also Gq protein (Fig. 2B). Thus, reduced GIP receptor signaling in β-cells due to altered signal amplification can also contribute to the reduced incretin effect under diabetic conditions. A study of Europeans using the glucose clamp technique showed that the incretin effect of GIP is rapidly reduced as fasting blood-glucose levels rise to 100 mg/dL [19]. However, the reduced incretin effect of GIP is recovered in those with type 2 diabetes once hyperglycemia has improved [20], indicating that the reduced incretin effect of GIP is reversible.

**Fig. 2:**
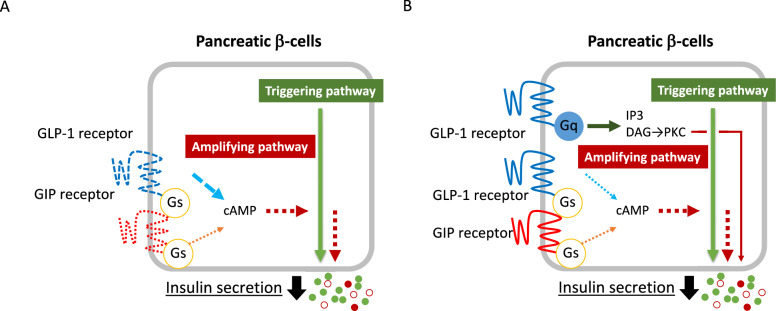
Causes of reduced incretin effect in pancreatic β-cells under diabetic condition. **A** Reduced expression of GIP and GLP-1 receptors on pancreatic β-cells, **B** change in amplification signaling from Gs protein dominance to Gq protein dominance. *IP3* inositol trisphosphate, *DAG* diacylglycerol, *PKC* protein kinase c, *cAMP* cyclic adenosine monophosphate, *Gs* Gs protein, *Gq* Gq protein

As the incretin effect of GLP-1 is preserved under diabetic conditions, incretin-based diabetes drugs (incretin-related drugs) have been developed, mainly targeting the action of GLP-1 [21]. However, GIP and GLP-1 are inactivated by dipeptidyl peptidase-4 (DPP-4) within minutes. The actions of GIP and GLP-1 are thus not sustained in vivo. GLP-1 receptor agonists have a structure resistant to DPP-4 and pharmacologically stimulate the GLP-1 receptor. While, DPP-4 inhibitors increase active GIP and GLP-1 levels by inhibition of DPP-4, enhancing physiologic GIP and GLP-1 action in vivo. Both drugs potentiate insulin secretion and inhibit glucagon secretion and strongly lower blood-glucose levels. In addition, GLP-1 receptor agonists act pharmacologically on GLP-1 receptors expressed in various organs and tissues. Therefore, a GLP-1 receptor agonists have several glucose lowering effects such as inhibition of gastric emptying and appetite suppression [22]. GLP-1 receptor agonists are also effective against various diseases such as fatty liver, cardiovascular disease, and chronic kidney disease. Thus, a GLP-1 receptor agonists are recommended for use in individuals with type 2 diabetes at high risk of cardiovascular disease and chronic kidney disease [23, 24]. Recently, tirzepatide, a new incretin-related drug, has become available for the treatment of type 2 diabetes. Tirzepatide is a GIP and GLP-1 receptor dual agonist and has a structure similar to human GIP (1–42) and exendin-4. This drug stimulates both GIP and GLP-1 receptors with a single peptide [25]. In vitro studies show that tirzepatide stimulates the GIP receptor more than the GLP-1 receptor [26, 27]. In a study using human islets, tirzepatide induced glucose-dependent insulin secretion [28]. In addition, a GIP receptor antagonist significantly reduced tirzepatide-induced insulin secretion compared to a GLP-1 receptor antagonist, indicating that tirzepatide promotes insulin secretion mainly through GIP signaling in β-cells.

With the advent of incretin-related drugs, various mechanisms of insulin secretagogue action by incretins have been elucidated. Glucagon and GLP-1 receptor dual agonists and GLP-1, GIP, and glucagon receptor triple agonists have been developed, and we will be able to use them for the treatment of metabolic diseases including type 2 diabetes, in future [29]. Glucose-lowering effects of GLP-1 receptor agonists and DPP-4 inhibitors are more effective in Asians than in Westerners [30, 31]. The results of clinical trials are awaited to determine the effects of these drugs on metabolic parameters including glucose tolerance in Asians.

## Abbreviations

cAMP: Cyclic adenosine monophosphate
DPP-4: Dipeptidyl peptidase-4
Epac2: Exchange protein activated by cAMP
GIP: Glucose-dependent insulinotropic polypeptide/gastric inhibitory polypeptide
GLP-1: Glucagon-like peptide-1
GPCR: G protein-coupled receptor
IVGTT: Intravenous glucose tolerance test
OGTT: Oral glucose tolerance test
PKA: Protein kinase A
VDCC: Voltage-dependent calcium channel
VIP: Vasoactive intestinal polypeptide
