# Correction: Regulation of glucose metabolism by incretins: implications for treatment of type 2 diabetes

**DOI:** 10.1007/s13340-025-00826-w

**Published:** 2025-06-09

**Authors:** Rie Saito, Norio Harada

**Affiliations:** Department of Endocrinology and Metabolism, School of Medical Sciences, University of Fukui, 23-3 Matsuoka Shimoaizuki, Eiheiji-cho Yoshida-gun, Fukui, 910-1193 Japan

## Correction: Diabetology International 10.1007/s13340-024-00781-y

The article “Regulation of glucose metabolism by incretins: implications for treatment of type 2 diabetes”, written by Rie Saito and Norio Harada, was originally published under exclusive license to The Japan Diabetes Society. As a result of the subsequent decision to publish the article under the open access model, the article’s copyright notice was changed on 17 May 2025 to © The Author(s) 2024 and the article is now distributed under a Creative Commons Attribution 4.0 International License, which permits use, sharing, adaptation, distribution and reproduction in any medium or format, as long as you give appropriate credit to the original author(s) and the source, provide a link to the Creative Commons licence, and indicate if changes were made. The images or other third party material in this article are included in the article’s Creative Commons licence, unless indicated otherwise in a credit line to the material. If material is not included in the article’s Creative Commons licence and your intended use is not permitted by statutory regulation or exceeds the permitted use, you will need to obtain permission directly from the copyright holder. To view a copy of this licence, visit http://creativecommons.org/licenses/by/4.0.

The original article has been corrected.

